# Asymmetric Lower Extremity Involvement and Facial Palsy: An Atypical Case of Guillain-Barre Syndrome

**DOI:** 10.7759/cureus.8912

**Published:** 2020-06-29

**Authors:** Anas Albawaliz, Dina Abdallah, Gurpreet K Seehra, Ahmed Elkafrawy, Ahmad Al-Shyoukh

**Affiliations:** 1 Internal Medicine, University of Missouri-Kansas City School of Medicine, Kansas City, USA; 2 Dentistry, University of Jordan, Amman, JOR; 3 Child Neurology, University of California, Los Angeles (UCLA), Los Angeles, USA; 4 Internal Medicine, University of Missouri-Kansas City School of Medicine / Saint Luke's Health System, Kansas City, USA

**Keywords:** guillain-barre syndrome, asymmetry, asymmetric, hyporeflexia, facial diplegia

## Abstract

Guillain-Barre syndrome (GBS) represents the most common cause of acute flaccid paralysis and is characterized by muscle weakness frequently accompanied by respiratory and bulbar paralysis which oftentimes can be life-threatening. Early recognition and intervention are essential to prevent potential complications and help hasten recovery. Herein, we report a case of a middle-aged female who presented with nonspecific gastrointestinal symptoms that were shortly followed by a unique combination of new-onset facial diplegia and asymmetric lower extremity areflexia. Treatment with intravenous immunoglobulins (IVIG) was initiated following a prompt diagnosis of GBS was made. Clinicians should always be vigilant about the possibility of GBS in the appropriate clinical setting and be aware of the essentials of management of this potentially treatable disease.

## Introduction

Guillain-Barre syndrome (GBS) is an acute paralytic polyneuropathy resulting from an autoimmune response directed towards peripheral nerves leading to heterogeneous forms of demyelinating and axonal damage. Several clinical variants of GBS have been recognized which can present with extremity, facial, respiratory, or bulbar muscle involvement [1]. Asymmetry in peripheral nerve involvement is atypical and has been only rarely described in the literature [2-6]. To our knowledge, combined facial diplegia and asymmetric lower extremity hyporeflexia is a unique presentation of GBS that our following case will serve to highlight.

## Case presentation

A 53-year-old female patient with a history of type II diabetes and hypertension presented with a one-week history of abdominal pain, constipation, nausea, and vomiting. She reported a single episode of bright red blood per rectum a few days prior to admission. Two days following the presentation the patient started complaining of perioral numbness, progressive bilateral facial weakness, and difficulty of swallowing initiation. On examination, vital signs were within normal limits. The patient was conscious, alert, and oriented. Cranial nerves’ exam was notable for diminished facial muscles' strength bilaterally (unable to smile or puff her cheeks, unable to maintain eyelid closure against resistance and unable to raise eyebrows bilaterally). Strength was preserved (5/5) in all proximal and distal bilateral upper and lower extremity muscle groups.

The patient had an absent left patellar reflex but otherwise had 2+ (normal) right patellar, and bilateral biceps, triceps, brachioradialis, and Achilles reflexes. The sensory exam was intact. The exam was also notable for a mildly distended abdomen but was otherwise unremarkable.

Constipation shortly resolved with laxatives and computerized tomography (CT) of the abdomen was unremarkable. With regard to the solitary episode of bloody stool, it was decided to proceed with colonoscopy as an outpatient. A stool culture was not sent as the patient presented with constipation rather than a diarrhea illness.

**Figure 1:**
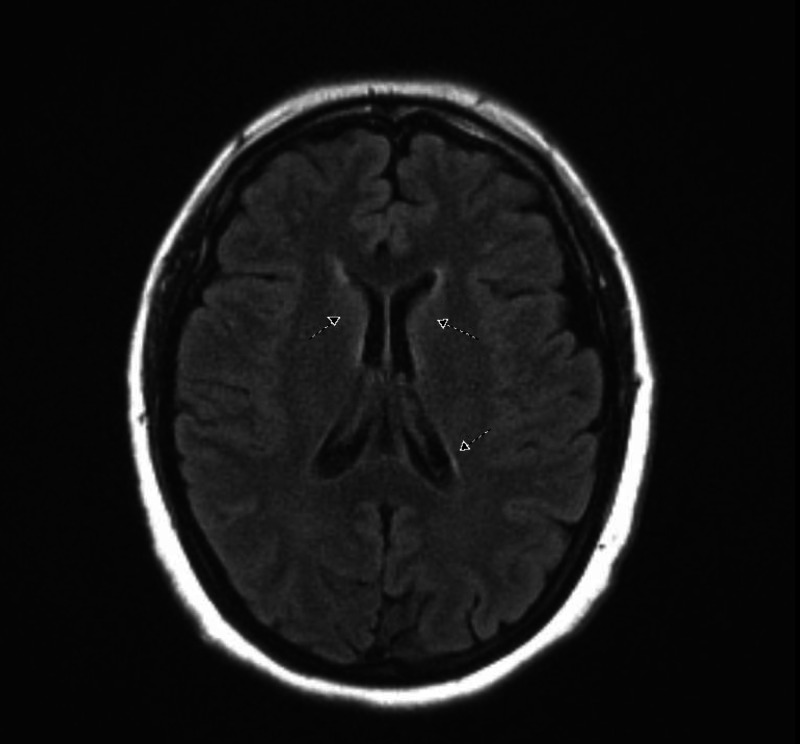
Magnetic resonance imaging of the brain. Mild nonspecific scattered subcortical and deep periventricular T2 FLAIR hyperintensities (arrows) suggestive of chronic small vessel ischemic disease.

Concerning her new neurological findings, CT and magnetic resonance imaging (MRI) (Figure 1) of the brain were obtained and were unremarkable except for changes of chronic small vessel ischemic disease. Neurology was consulted who raised the concerns of an atypical presentation of Guillain-Barré syndrome and recommended cerebrospinal fluid (CSF) examination. Same day lumbar puncture (LP) demonstrated increased protein (93 mg/dL) with absent white and red blood cells. No microorganisms were visible on gram stain. Due to the high suspicion of GBS, the patient was urgently started on intravenous immunoglobulins (IVIG) with a dose of 400 mg/kg/day. The level of care was escalated to the progressive care unit where the patient underwent frequent negative inspiratory force (NIF) and forced vital capacity (FVC) monitoring which remained within normal limits.

The patient completed a five-day course of IVIG during which she had no worsening of symptoms but only a slight improvement. On day 9 of hospitalization, the patient decided to leave against medical advice (AMA) and did not show up for her follow-up appointment.

## Discussion

Guillain-Barré syndrome is an autoimmune demyelinating disorder usually heralded by a gastrointestinal or respiratory tract infection that incites an abnormal immune response targeting peripheral nerves [1,7]. Among the first clinical manifestations of GBS are pain, numbness, paresthesia, and/or weakness in the limbs. Weakness usually presents in the distal extremities in a rapidly progressive, ascending, and symmetric fashion. However, it can also begin more proximally in the legs or arms. Many patients develop reduced tendon reflexes in the affected limbs [8-10]. About half of the patients present with cranial nerve deficits, particularly bilateral facial weakness, swallowing difficulties (bulbar symptoms), and/or oculomotor dysfunction, which might later extend to involve the limbs [8,11].

The combination of rapidly progressive symmetrical weakness with or without sensory disturbances, hyporeflexia, or areflexia in the absence of a CSF cellular response but elevated protein level remains the hallmark for the clinical diagnosis of GBS. Identification of symptoms and establishing diagnosis need to be made promptly to avoid the devastating sequelae of untreated disease which can result in respiratory muscle paralysis. The appropriate level of care with close observation and frequent monitoring of negative inspiratory force (NIF) and forced vital capacity (FVC) are necessary once the diagnosis is made [1].

Early administration of intravenous immunoglobulins (IVIG) or initiating plasma exchange (PLEX) are the mainstay treatment, particularly in patients with rapidly progressive weakness [12]. Without treatment, symptoms continue to progress for up to two weeks and slowly plateau over 2-4 weeks with subsequent gradual recovery of function in the majority of patients. Disease-modifying treatment helps shorten this timeline [13,14].

Our described case illustrates how variable GBS presentation can be. Asymmetry in extremity involvement is only rarely described in the literature [2-6]. Further, the unique combination with facial diplegia makes the presentation even more atypical. However, due to suspicious presentation, prompt CSF analysis was obtained which confirmed the diagnosis. Timely IVIG administration alongside supportive care helped to avoid potential respiratory complications.

## Conclusions

GBS is the most common and most severe acute paralytic neuropathy that can present in several forms. Clinicians should have a low threshold for diagnosis in the appropriate clinical setting which should prompt immediate intervention. Proper management decreases the likelihood of progression to respiratory failure and shortens the recovery period. This case discusses an atypical presentation of GBS which would help physicians be more vigilant about diagnosing and treating such cases as early as they present.
